# Improving access to safe water and groundwater sustainability

**DOI:** 10.1016/j.lansea.2025.100558

**Published:** 2025-03-10

**Authors:** The Lancet Regional Health – Southeast Asia

On World Water Day, observed on March 22, we need to reflect on the goal of ensuring safe water for all by 2030. March marks the onset of summer in several countries within the WHO South-East Asia Region (SEAR), and in India, it also signifies the beginning of a water crisis. Every summer, there is an initial public outcry, but the water crisis is often forgotten when the monsoon arrives in June, perpetuating the cycle. Although 80% of India receives rainfall, the uneven distribution across states leads to heavy dependence on groundwater. However, the increasing, unsustainable water demand in recent years is gradually depleting groundwater levels. In 2022, only 93% of Indians had access to water (within a maximum round trip of 30 min from home to the source). Access to clean water (Sustainable Development Goal 6) remains a recurring issue in election manifestos.

Due to India's topography, rainfall, and other environmental factors, each Indian state experiences a different climate, with hotter states being the most affected. Although access to water is a fundamental right, it often becomes a privilege due to socioeconomic disparities. The water crisis predominantly affects rural areas due to the lack of piped water. The states of Kerala and Uttarakhand have the highest scores for progress related to the Sustainable Development Goals. Still, much of the population in Kerala lacks basic amenities such as a tap water supply, access to drinking water, and water for use in toilets. As of October 2024, India's *Jal Jeevan Mission* has provided tap water to 119.5 million rural households. Despite several schemes in place to address the urgent water shortage, there is a lack of political will to maintain these systems effectively. Consequently, taps are dry, and pipes are broken.

India is the world's largest groundwater user, with a massive 250 km^3^ of groundwater being used by Indian farmers for irrigational purposes. This quantity is more than the combined usage by China and the USA. The northern and central part of India has more unsustainable water demand due to cultivation of wheat, rice, and sugarcane, Shortage in rainfall leads people to employ methods like borewells, which, if unregulated, can result in ecological devastation and exploitation of groundwater. The number of borewells in India increased from 1 million to 20 million during the last five decades. In contrast, farmers—instead of constructing new wells—are further digging old wells, indicating the gravity of the water crisis. Previously in the agricultural state Punjab, farmers were given free rein to construct borewells. Even though the water table would usually be accessible at a depth of 50–60 feet, farmers in Punjab now struggle to find water even at 150–200 feet. *Atal Bhujal Yojana (ATAL JAL)*, the Indian Government's scheme established in 2020 to improve groundwater sustainability, is planned to extend to 12 states including Tamil Nadu, Punjab, Telangana, Bihar, and Andhra Pradesh. The Government has claimed a reduction in groundwater extraction from 249 billion cubic metres (bcm) in 2017 to 245.64 bcm in 2024. Unfortunately, this is only a 1.35% reduction in 7 years. There is still more to do.

In some Indian states, groundwater crises and access to safe water are pressing issues, especially in rural areas. Industries, with vested interests and political support, are sometimes allowed to exploit local water bodies. Industrial effluents are released into water bodies without fear of disciplinary action. Extensive use of fertilisers by farmers and leaching from geological sources have also compounded the issue. The time spent collecting safe water from distant sources hinders people's productivity and prevents them from improving their economic status. Hence, the time invested in the collection and maintenance of safe water drives more people into poverty. According to the Annual Ground Water Quality Report 2024, high levels of uranium, fluoride, chloride, arsenic, and nitrates have negatively impacted the potability of groundwater in several districts of Punjab and Haryana. A 2024 study showed that children and adults living near the Majha Belt of Punjab were at higher health risk due to arsenic contamination in drinking water. Increasing urbanisation, without proper wetland management, has led to the over-exploitation of water bodies and the destruction of fragile ecosystems. Political will is a major solution. For example, the Hyderabad Disaster Response and Assets Protection Agency (HYDRAA) demolished several lake encroachments near Hyderabad, and is on course to rejuvenate 1025 lakes. However, HYDRAA has been criticised for not providing adequate time to the residents before proceeding with the demolitions. Increased power to environmental protection bodies can help prevent environmental injustice.

Desalination of seawater could be a potential solution to the water crisis. For example, 60% of Saudi Arabia's drinking water is desalinated seawater. However, the high operational costs of the desalination process make its sustainability a challenge for low- and middle-income countries in SEAR. Another challenge is brine discharge, a by-product of desalination, which must be carefully released back into the sea at various locations to avoid harming marine flora and fauna. On a positive note, desalination plants are steadily increasing in Asia. For example, several populated islands of Maldives have dedicated desalination plants, and new projects are ongoing in Thailand and India.

Another solution is wetland management. Over the past three decades, China has made commendable efforts in this area. In 1992, China joined the Ramsar Convention, an international treaty for the conservation and sustainable use of wetlands. Following three national surveys conducted over many years, China designated 1021 Wetlands of Provincial Importance, 29 Wetlands of National Importance, and 64 Wetlands of International Importance. The protection of these wetlands was achieved through various laws and public awareness initiatives. Another success story is the restoration of Polachery Lake, 35 km from Chennai, India, which involved bund construction, recharge ponds, and invasive weed removal, with crucial support from residents.

Countries in SEAR must promote water, sanitation, and hygiene (WASH), especially the effective disposal of child faeces, to prevent water contamination. Active water sampling and monitoring are required to avoid preventable deaths. Governments must also be involved in data collection related to inequalities in safe water, which would help them make evidence-based decisions, allocate funds, support innovations, and build new infrastructure. To improve groundwater levels, both central and state governments can actively promote crop rotation and provide canal irrigation to farmers. Governments of countries with access to seawater must encourage new innovations, promote the establishment of desalination plants via subsidies, and conduct cost-effectiveness studies. Using more wind and solar power in the desalination process could help lower the costs eventually.

Investing in clean water will uphold human dignity. We want everyone, especially women and children, to have more time for themselves rather than collecting water. We also want future annual reports to publish lower numbers related to the inaccessibility of safe water. With strong political commitment and collaborations among communities, we can move forward.

